# T lymphocytes and normal tissue responses to radiation

**DOI:** 10.3389/fonc.2012.00119

**Published:** 2012-09-19

**Authors:** Dörthe Schaue, William H. McBride

**Affiliations:** Department of Radiation Oncology, David Geffen School of Medicine, University of California, Los AngelesLos Angeles, CA, USA

**Keywords:** radiation, T cells, Tregs

## Abstract

There is compelling evidence that lymphocytes are a recurring feature in radiation damaged normal tissues, but assessing their functional significance has proven difficult. Contradictory roles have been postulated in both tissue pathogenesis and protection, although these are not necessarily mutually exclusive as the immune system can display what may seem to be opposing faces at any one time. While the exact role of T lymphocytes in irradiated normal tissue responses may still be obscure, their accumulation after tissue damage suggests they may be critical targets for radiotherapeutic intervention and worthy of further study. This is accentuated by recent findings that pathologically damaged “self,” such as occurs after exposure to ionizing radiation, can generate danger signals with the ability to activate pathways similar to those that activate adoptive immunity to pathogens. In addition, the demonstration of T cell subsets with their recognition radars tuned to “self” moieties has revolutionized our ideas on how all immune responses are controlled and regulated. New concepts of autoimmunity have resulted based on the dissociation of immune functions between different subsets of immune cells. It is becoming axiomatic that the immune system has the power to regulate radiation-induced tissue damage, from failure of regeneration to fibrosis, to acute and chronic late effects, and even to carcinogenesis. Our understanding of the interplay between T lymphocytes and radiation-damaged tissue may still be rudimentary but this is a good time to re-examine their potential roles, their radiobiological and microenvironmental influences, and the possibilities for therapeutic manipulation. This review will discuss the yin and yang of T cell responses within the context of radiation exposures, how they might drive or protect against normal tissue side effects and what we may be able do about it.

## Introduction

Lymphocytes are a common component of the leukocyte infiltrates found in irradiated tissues both during acute and chronic phases (Hong et al., 2003; Chiang et al., 2005; Fajardo, 2005; Yoshii, 2008; Moravan et al., 2011), and in humans and other species (Teymoortash et al., 2005; Toma et al., 2010). While some pathologists insist that they are unimportant as a cause of radiation-induced damage (Fajardo, 2005), many consider them as contributing to radiation-induced symptoms and pathogenesis. Strangely, both may be right! However, many lymphocytes in irradiated lesions seem activated rather than innocently trapped and since activated lymphocytes function as effector cells and/or as managing directors of bystander non-immune and immune cells through the release of effector cytokines, it is hard to ignore their significance. In this sense, acute and late radiation effects can be considered as acute and chronic inflammatory diseases. Placing lymphocytes in the correct framework to understand their possible roles; however, requires understanding of immune recognition of “self” and how responses are controlled by allocating immune functions to different T cell subsets and this will be the focus of this review, with the understanding that other lymphocyte subsets are present and may also be important.

The need to transplant tissues to help casualties during the Second World War foundered on what Medawar called the “uniqueness of the individual.” The phenomenon of transplant rejection, however, fostered important immunological theories based on recognition of “self”/“non-self.” Consensus grew for antigen-driven selection and expansion of responsive lymphocyte clones that developed so as to express a single specificity. Positive and negative selection processes during development were postulated to shape the immune repertoire so as to eliminate the chance of autoimmunity. These theories have generally stood the test of time. T cell repertoires are shaped in the thymus by positive selection of naive T cell clones with T cell receptors (TCR) that have moderate affinity for self MHC antigens and that later develop into effector T cells recognizing peptide antigen in peripheral lymphoid organs in the context of MHC class I (CD8+ cells) or class II (CD4+ cells). Negative selection allows those with low affinity receptors for MHC+self to move on while those with high affinity for “self” get deleted, probably by more than one mechanism (central tolerance). These selection processes are, however, far from complete and the concepts have had to be modified to invoke mechanisms to maintain tolerance when it became clear that anti-self reactivity was still possible (peripheral tolerance). This is largely the purview of Tregs, whose absence causes severe autoimmune disease, excessive lymphoid proliferation, and immune destruction that is basically incompatible with life. Tregs (formally known as suppressor T cells) are the major players in preventing excessive damage to self (Peterson, 2012), and there is every reason to assume that they play important roles in radiation-induced tissue damage.

## T cell subsets

CD4+ naïve T cells have the capacity to differentiate along different paths so as to effect distinct functions following antigenic stimulation largely through elaboration of distinct cytokine profiles. Initially, mutually exclusive Th1 and Th2 subsets helped explain why cellular immunity (Th1-mediated) could exist in the absence of antibody production (Th2-mediated) and vice-versa. More recently, the Th17 subset, which is a potent inducer of tissue inflammation and has been associated with the pathogenesis of many autoimmune diseases, has emerged, as have adaptive or induced Tregs (iTregs) that share participation of TGF-β in their differentiation. Also, a Tfh subset of follicular helper cells assists B cells in antibody production. All these “classic” T cells become effector cells by engagement of TCRs containing α and β chains (signal 1) and of co-stimulatory molecules (signal 2) expressed by mature antigen presenting cells, which requires specific cytokines produced primarily by innate immune cells that recognize molecules in a pathological site through pattern recognition receptors (PRRs). Dendritic antigen presenting cells (DC) appear to be the major drivers of these responses, but there are others. Each T cell subset has distinct transcription factors controlling their functionality and they often appear to operate in a reciprocal fashion. One way this can occur is through interconversion for example of iTregs into Th17 by IL-6. Th1 and Th2 subsets are less flexible though they often appear to be in opposition. The factors that drive specific T cell subsets are the keys that unlock the enormous therapeutic of the immune system.

In addition to these adaptive T cell subsets, natural subsets of regulatory cells (nTregs), γδT cells, and natural killer T (NKT) cells emerge from the thymus ready for action and bridge innate and adaptive immunity. nTregs, like iTregs are controlled by the Foxp3 transcription factor. They produce immunosuppressive cytokines like TGF-β and IL-10 and both have TCRs that focus on “self” antigens. Cells bearing TCRs composed of γ and δ chains mostly lack CD4 and CD8 expression and are not MHC restricted. They respond primarily to small non-peptide phosphoantigens, which are metabolites of isoprenoid biosynthesis pathways. While they are found in blood, they form a more major component of the intra-epithelial lymphocyte pool. They are able to produce Th1-type cytokines like IFN-γ and TNF-α and may be a major innate source of IL-17. The exact distinctions between γ δT cells, Th1/Th17, and nTh17 cells that are reported in the literature are not yet clear. γ δT cells, however, form a first line of defense against Mycobacteria and Candida, as well as being involved in anti-tumor responses and autoimmunity. They have been found to accumulate around blood vessels and mucosal airways in macaques that have inhaled *Yersinia pestis* (Huang et al., 2009), where they release the homeostatic cytokine FGF-7. This is reminiscent of perivascular cuffs of lymphocytes often seen in radiation-damaged tissues. NKT cells, in contrast, express an invariant and limited TCR that recognizes lipid antigens presented in the context of CD1d. They play diverse roles in enhancing some forms of cell-mediate immunity while being more suppressive toward autoimmune responses.

The prelude to the adaptive T cell overture involves more than just innate T cells. Ionizing radiation like other insults damages tissues, and damaged tissues show and tell various “danger” signals including Damage-Associated Molecular Pattern molecules (DAMPs) to the immune system (Matzinger, 2002; Shi et al., 2003; Lotze et al., 2007; Curtin et al., 2009; Sato et al., 2009; Kawai and Akira, 2011). DAMPs can be secreted and/or released into extracellular spaces prior to cell death but the most dramatic surge follows cell death with the release of HMGB1, dsDNA, chromatin, RNA, mitochondria, etc. It may be significant that the conformation of intracellular molecules may change when they are in an extracellular space with oxidation making such moieties a particularly interesting source of radiation DAMPs (Miller et al., 2011). Once released, DAMPs bind to PPRs and initiate signaling cascades and communications between immune cells through activation of cytokine and chemokine networks so as hopefully to eliminate danger and restore homeostasis, leading to regeneration and healing of tissues (Schaue and McBride, 2010; Schaue et al., 2012). This process is characterized by infiltration of various host cells into the site—a textbook inflammatory response—initially polymorphs and monocytes and slightly later lymphocytes. Alterations in cellular subsets and functions with time aim to turn the pro-inflammatory, pro-oxidant environment into one that is more compatible with tissue restoration and rescue. In the case of radiation-damaged mucosal surfaces, various DAMPs may actually work in cohort with microbes to generate inflammatory infiltrates and activate innate immune defenses (Abreu et al., 2005). A critical issue is whether the irradiated microenvironment is sufficient to mature DCs into competent antigen presenting cells that can activate adaptive T cell responses (Banchereau and Steinman, 1998; Gallucci et al., 1999). In other words, DAMPs initiate signaling cascades in DCs that drives them to not only present the antigen to T cells (signal 1) but simultaneously to mature and to show co-stimulatory molecules (signal 2), both of which are needed to kick off a full-blown immune response. Conversely, antigen-presentation in the absence of danger leads to a muted or anergic response as T cells have been taught to ignore or are switched off by any antigen that is not shown in the right context (Steinman et al., 2003).

The important concept that emerges is that “danger” tunes-up the immune system to proceed in more orderly progressions to develop a symphony involving multiple elements with feedback through chemokine and cytokine control loops that amplify and reiterate themes until final resolution. Adaptive immune effector T cells play major roles in perpetuating damaging processes while Tregs and other suppressor cells calm the situation and try to bring resolution. As a result, examination of an immune reaction site may show a predominance of a particular T cell subset and associated cytokine profile at any one time, but at least some of the rest of the orchestra will inevitably be present with the relative contributions varying with time. Perhaps the most surprising and exciting discovery in recent years is that the volume and the nature of the response is under tight control primarily by Treg cells and that their removal uncovers an immune system that is able to participate in multiple forms of autoimmune responses, indicating the extent to which central tolerance is limited as a mechanism for controlling autoimmunity. The corollary is that the immune system is in “lock down” mode under most normal circumstances and the generation of an effector response requires local removal of restrictions. These concepts are highly relevant for consideration of the role of T cells in radiation-induced normal tissue responses, just as they are for immune recognition of tumors during radiation therapy.

## Signs of autoimmunity after tissue irradiation

Damage to tissues following irradiation is a result of direct cell kill plus collateral or bystander damage caused by vasculature damage and inflammatory infiltrates. Initially, changes in redox in the irradiated normal tissue microenvironment play a role in preparing it for immune recognition and activation. This involves decorating it with recognition molecules and release of chemokines and chemokines and receptors that attract different inflammatory subsets cells (Lorimore et al., 2001; Lugade et al., 2005, 2008; Matsumura et al., 2008; Burnette et al., 2011). We, and many others, have shown that irradiation increases expression of MHC class I and II molecules, co-stimulatory molecules, chemokine receptors, and other cell surface markers (Santin et al., 1996; Morel et al., 1998; Seo et al., 1999; Garnett et al., 2004; Reits et al., 2006; Tyurina et al., 2011). The pro-inflammatory, pro-oxidant milieu that develops in tissues in the aftermath of irradiation is therefore one that encourages immune recognition and that comes with the possibility of autoimmunity.

Historically, autoimmunity was considered to be an aberrant response. More recently, it has been recognized as a normal condition with cells with potential self-reactivity existing in everyone, even though autoimmune disease *per se* is relatively uncommon, afflicting 5–8% of the population in the USA. The question is whether radiation treatment generates anti-self reactive responses, what types of response are generated, and whether they play a role in normal tissue effects. Unfortunately such responses are easy to observe only when they cause disease, which involves a degree of genetic susceptibility though this is not as important as environmental factors. Autoimmune disease after radiation therapy may, however, develop more readily in people with a family history of autoimmunity and radiation may have a more detrimental effect in such a subpopulation. A case in point is the possible link between autoimmune celiac disease and susceptibility to radiation pneumonitis that seems to exist (Eriksen et al., 2012). In all these discussions, the extent of the radiation field and dose are important considerations for how irradiation alters the balance within the immune system and how it may drive autoimmune recognition.

The literature on radiation and autoimmunity is quite extensive. Mice prone to autoimmune thyroiditis respond to whole-body irradiation (WBI) with a higher incidence and higher severity of the disease and higher anti-thyroglobulin autoantibody titers than the wild type (Nagayama et al., 2009). Humans whose thyroids are exposed to radiation in therapeutic and environmental settings also develop elevated autoantibodies at least for a window of time (Brent, 2010). Other examples of radiation-induced autoimmunity exist. Morphea, a rare but painful skin complication in women following breast cancer radiotherapy may be in fact due to radiation-induced neoantigen formation (Laetsch et al., 2011). In a study of sialadenitis of the submaxillary gland in head and neck cancer patients following radiation therapy, it was concluded that acinar cell destruction was associated with an influx of cytotoxic T cells (Teymoortash et al., 2005). In the lung, lymphocytic alveolitis is a marker of radiation pneumonitis in breast cancer patients receiving radiation therapy (Toma et al., 2010) and T cells can readily be isolated from bronchoalveolar lavage in patients after lung irradiation (Nakayama et al., 1996). Even radiation doses that do not cause any obvious lung damage can elevate T cell numbers in bronchoalveolar lavage fluids in mice for a long time (Johnston et al., 2011). It is not surprising that radiation-induced interstitial pneumonitis and alveolitis is a common complication in syngeneic and allogeneic bone marrow transplantation (Shankar et al., 1999; Beyzadeoglu et al., 2004) and that this can be exacerbated by T cell responses to infectious agents (Bruckner et al., 2006). Cyclophosphamide given in combination with WBI also increases the damage to the lung and there is an inverse relationship with bone marrow damage (Yan et al., 1991). This reciprocal relationship might in part be because some of the radiation-induced lung damage is in fact immune-mediated, i.e., it needs the supply of immune players from the bone marrow. In keeping with this concept, thymic reprocessing seemed to enhance the incidence of radiation-induced pneumonitis in mice as it was decreased by thymectomy of mice receiving WBI and syngeneic bone marrow transplantation; an effect that was reversed by spleen cell delivery (McBride and Vegesna, 1997, 2000). The ability of radiation to cause autoimmunity was emphasized by the finding that very high-dose, fractionated, total lymphoid irradiation (TLI)-induced multi-organ autoimmunity in mice, with characteristics dependent on radiation dose, extent of lymphoid irradiation, and the genetic background (Sakaguchi et al., 1994). This contrasts with studies where irradiation has been used to suppress autoimmunity.

## Suppression of autoimmunity after tissue irradiation

Despite the examples of radiation-induced autoimmune reactions cited above, remarkably, WBI or TLI followed by autologous stem cell transplantation has been used to treat already-established spontaneous autoimmune diseases such as systemic lupus erythematosus or rheumatoid arthritis with the aim of eliminating autoreactive lymphocytes (Loor et al., 1988). Astonishingly, this is successful in a high proportion of patients despite the fact that depletion of autoreactive cells is never complete and there is always a possible contribution to failure from T cells in the autologous graft. Homeostatic expansion of Tregs may contribute but success seems to be mostly due to thymic-dependent regeneration of Tregs and reprogramming of the imbalanced immune networks following irradiation (van Wijk et al., 2008). This may take 9 months to occur in humans and there appears to be a window of time when tolerance can be re-established, with ongoing chronic inflammation and high levels of danger signaling being negative long-term predictors for success. In mice, low repeated doses of WBI attenuated autoimmune allergic encephalitis by up-regulating Tregs and suppressing IL-17 production (Tsukimoto et al., 2008), and appears to require recognition of TNFR2 on non-hematopoietic cells by Tregs (Tsakiri et al., 2012).

Similar immunosuppressive reprogramming can follow TLI. This was introduced in the late 1970s by Kaplan and Strober and later shown to be due to the generation of suppressor cells (Waer et al., 1984). Even patients with graft failure or graft rejection after allogeneic hematopoietic stem cell transplantation can be rescued by TLI-based reconditioning regimens resulting in sustained engraftment (Heinzelmann et al., 2008). The suppressor cells in the early preclinical studies of LTI and WBI were induced independent of the thymus (Weigensberg et al., 1984) and may have been myeloid or NKT cells, although the addition of anti-thymocyte serum to LTI has more recently documented Treg cell involvement (Nador et al., 2010). The explanation for the apparent discrepancy in radiation both causing and treating autoimmunity may lie in whether generalized immune reprogramming is occurring, whether the irradiation is localized or extensive, the radiation dose, the state of the immune system at the time of the exposure, i.e., in suppressor or effector mode, and the extent and nature of the “danger” signals that are generated. The immune system is normally in balance and this can be tipped in more than one direction depending on the therapeutic intervention. This will also change with time as different subsets take different times to regenerate. “Rebalancing” of course means something rather different if one is treating autoimmune disease as compared with cancer treatment or after a radiological incident. The important issue for the future is understanding the pathways involved so that they can be manipulated appropriately for the given situation.

## Radiation kills lymphocytes selectively

Radiation is often viewed as an immune-suppressive agent and most lymphocytes are indeed very radiation sensitive. Even local RT will have a direct cytotoxic effect on the circulating immunocyte pool as blood flows through the field, irrespective of other, more complex effects associated with immune activation. The extent of immune depletion will vary with the tissue, the size of the field, the delivery schedule, and dose (MacLennan and Kay, 1978). Cranial irradiation, for instance, can cause long-term lymphopenia in children to an extent that depends on the number of fractions i.e., dose (MacLennan and Kay, 1978). In reality, however, lymphocyte subsets differ in their radiation sensitivities. This is most obvious in lethal irradiation for bone marrow preconditioning, which does not eliminate all lymphocytes equally (Bagley et al., 2002). In very general terms, a spectrum of radiosensitivity exists from B cells through naive Th cells, NK cells, toward more radioresistant T memory cells (Belka et al., 1999) and NKT cells (Yao et al., 2011), and Tregs (Kachikwu et al., 2011). There is a tendency toward apoptosis denoting a more radiosensitive phenotype, while non-proliferative cells and activated lymphocytes are more radioresistant (McBride et al., 2004), although Tregs probably gain in radiosensitivity when they are induced (Awwad and North, 1988).

The end result is that irradiation imbalances the immune system. This may be restored with time depending upon the recovery time for the different immune subsets, but may also persist for long periods or even forever as is seen in A-bomb survivors. Since the distribution of T cell subsets is organ-specific (Matzinger and Kamala, 2011), recovery will proceed at different rates in different tissues and will depend on the chemokines produced (Pelus and Fukuda, 2008). An extreme example of this is recovery of γδTCR CD4+ intraepithelial T cells in the gut after WBI. This is independent of the thymus and occurs very rapidly in mice, while αβTCR T cells subsets take several weeks to recover (Mosley and Klein, 1992). In this analysis, the relative radiosensitivity and recovery of Tregs following local RT and WBI would be expected to be rapid if this mechanism protects against radiation-induced normal tissue damage.

## Tregs and normal tissue radiation responses

Recently, Tregs have been shown to be relatively radioresistant and their representation increases rapidly following irradiation, as might be expected if they are involved in limiting damage and associated inflammation (Cao et al., 2009; Kusunoki et al., 2010; Nakatsukasa et al., 2010; Qu et al., 2010; Weng et al., 2010; Billiard et al., 2011; Kachikwu et al., 2011). Further induction and activation may be through the powerful immune-suppressive cytokine TGF-β (Chen et al., 2003; Beal et al., 2012; Takahashi et al., 2012) that is induced by RT (Martin et al., 2000), but additional mechanisms are possible. We have shown radiation-enhanced expression of the ectonucleotidase CD39 on the Treg population (Schaue, unpublished), that was previously observed in cancer patients receiving RT (Mandapathil et al., 2009). Nucleotide catabolism by CD39 and CD73 with production of adenosine is probably the most primitive immunosuppressive response to “danger.” Adenosine has long been known to play a critical, non-redundant role in the protection of normal tissues from collateral damage during inflammation (Cronstein, 1994), including radiation-induced tissue damage (Hosek et al., 1992; Pospisil et al., 1993, 1998; Hou et al., 2007), where it plays a protective role (Hofer et al., 2002). Support for this scenario comes from the observation that tissue derived adenosine acting through its receptor A_2A_R drives Tregs and limits autoimmune tissue destruction (Zarek et al., 2008).

Importantly, Treg radiosensitivity seems to depend upon their subtype and proliferative/activation status. This would explain the discrepancy seen between recent findings of radio-resistant Tregs and the radiation-sensitive Tregs first reported over 30 years ago (Hellstrom et al., 1978; Tilkin et al., 1981; North, 1986). Then, sublethal, WBI was used to eliminate suppressor T cells and uncover immune-mediated mechanisms leading to tumor regression. The timing of the radiation exposure post-tumor implantation was critical suggesting that a Treg subpopulation had been induced that was sensitive to radiation. Although Tregs have a reputation for being relatively anergic to antigenic stimulation *in vitro*, they appear to be able to proliferate *in vivo* following stimulation (Walker, 2004), which might make them radiosensitive. The type of Treg may also be important. The majority of Tregs are naturally occurring nTregs that come from the thymus. Induced Tregs can arise outside the thymus from peripheral conversion of CD4^+^CD25^−^ naïve T cells. Induction can be a result of exposure to low doses of antigen, IL-2, and TGF-β (Apostolou and von Boehmer, 2004; Curotto de Lafaille et al., 2004). So far it has remained difficult to definitively distinguish nTregs from iTregs, phenotypically and functionally, but it is clear that they do not have the same workload when it comes to controlling adaptive immune responses. There is some evidence that iTregs exert control of inflammatory responses at normal mucosal surfaces while nTregs appear more important for mediating self tolerance and tumor immune escape (Sakaguchi, 2004, 2005; Curotto de Lafaille and Lafaille, 2009; Haribhai et al., 2011; Rosenblum et al., 2011; Josefowicz et al., 2012). The differential sensitivity of nTregs and iTregs and their proliferative versus non-proliferative state could go a long way toward explaining some of the divergent data emanating from studies on the effects of RT on autoimmunity and anti-tumor responses that are described above, although further studies are urgently needed to elucidate the critical variables in these systems.

Taken together, the idea that radiation drives Tregs to protect tissues against radiation damage seems plausible. Recent findings that Tregs regulate the hematopoietic stem cell niche (Urbieta et al., 2010) suggest an even more important role for these cells in maintaining microenvironments that are protected from pro-inflammatory influences and allowing them to regenerate after irradiation. The impairment in Tregs in acquired aplastic anemia is in keeping with this concept (Shi et al., 2012). At the same time, the ability of HIF-1α to drive Th17 cells toward differentiation and pro-inflammatory autoimmune responses (Pan et al., 2012), as can IL-6 and adenosine (Wilson et al., 2011), suggests ways in which the tissue microenvironment that is generated by irradiation might negate the influence of Tregs (Radhakrishnan et al., 2008). Other cytokines such as TGF-β, IL-1, IL-23, and TNF-α will also influence these processes (Schaue et al., 2012). The concept seems to be evolving that the balance between the normal tissue microenvironment induced by irradiation and the immune system is decisive in controlling the extent of damage and regeneration or replacement of tissue following exposure, and the degree of local immunity that is generated.

## Intercepting immune mechanisms to protect normal tissues

The picture that is emerging draws a line from radiation-induced tissue damage, the release of danger signals all the way to inflammation and the activation of the immune system and its immune control mechanisms, to tissue regeneration (Schaue and McBride, 2010). The immune cells that infiltrate the damaged area in the immediate aftermath of irradiation, as well as those cells that were affected directly during the radiation exposure will determine the ultimate fate of that tissue, with influence from the genetic make-up of the host and multiple tissue-specific factors. What is lacking is information regarding the extent of polarization within the T cell and other cellular compartments and the associated cytokines they release that we associate with various characteristic normal tissue side effects such as pneumonitis and fibrosis in the lung, demyelination, gliosis and vascular damage in the brain, erythema, desquamation, fibrosis in the skin, diarrhea, ulceration, and other intestinal dysfunctions (Brush et al., 2007; Linard et al., 2012; Schaue et al., 2012). Genetics will play a role as will standard radiobiological parameters of dose, dose rate, volume, etc. Defining the irradiated tissue microenvironment in these terms will be important if we wish to utilize the numerous tools that could be of value in radioprotecting normal tissues, mitigating radiation-induced damage, and treating patients with complications from RT. Tools to target pathways involving chemokines and cytokines like TNF-α, IL-1, CSFs, IL-6, IL-17, TGF-β, HIF-1, cell adhesion molecules, and receptors like ICAM-1, CD11b, TLRs, transcription factors like HIF-1, NF-κB, p53, STATs, as well as T cell subsets and immune modulatory molecules, are currently in the clinic or in the pipeline. Conceptually, the targets are fairly obvious, but target validation is generally poorly developed for radiation-induced normal tissue reactions. There are risks in trying to manipulate immune control mechanisms as was seen with TGN1412 (Hunig, 2012), however, the potential is great and the prize could be a major increase in radiotherapeutic benefit in cancer treatment and ways to improve the outcome of radiological accidents and incidents.

### Conflict of interest statement

The authors declare that the research was conducted in the absence of any commercial or financial relationships that could be construed as a potential conflict of interest.

## References

[B1] Abreu M. T. Fukata M. Arditi M. (2005). TLR signaling in the gut in health and disease. J. Immunol. 174, 4453–4460 15814663 10.4049/jimmunol.174.8.4453

[B2] Apostolou I. von Boehmer H. (2004). *In vivo* instruction of suppressor commitment in naive T cells. J. Exp. Med. 199, 1401–1408 10.1084/jem.20040249 15148338 PMC2211808

[B3] Awwad M. North R. J. (1988). Sublethal, whole-body ionizing irradiation can be tumor promotive or tumor destructive depending on the stage of development of underlying antitumor immunity. Cancer Immunol. Immunother. 26, 55–60 2964269 10.1007/BF00199848 PMC11038398

[B4] Bagley J. Tian C. Sachs D. H. Iacomini J. (2002). T cells mediate resistance to genetically modified bone marrow in lethally irradiated recipients. Transplantation 74, 1454–1460 10.1097/01.TP.0000034625.87602.3B 12451248

[B5] Banchereau J. Steinman R. M. (1998). Dendritic cells and the control of immunity. Nature 392, 245–252 10.1038/32588 9521319

[B6] Beal A. M. Ramos-Hernandez N. Riling C. R. Nowelsky E. A. Oliver P. M. (2012). TGF-beta induces the expression of the adaptor Ndfip1 to silence IL-4 production during iT(reg) cell differentiation. Nat. Immunol. 13, 77–85 10.1038/ni.2154 22080920 PMC3542978

[B7] Belka C. Ottinger H. Kreuzfelder E. Weinmann M. Lindemann M. Lepple-Wienhues A. Budach W. Grosse-Wilde H. Bamberg M. (1999). Impact of localized radiotherapy on blood immune cells counts and function in humans. Radiother. Oncol. 50, 199–204 10368044 10.1016/s0167-8140(98)00130-3

[B8] Beyzadeoglu M. Oysul K. Dirican B. Arpaci F. Balkan A. Surenkok S. Pak Y. (2004). Effect of dose-rate and lung dose in total body irradiation on interstitial pneumonitis after bone marrow transplantation. Tohoku J. Exp. Med. 202, 255–263 15109123 10.1620/tjem.202.255

[B9] Billiard F. Buard V. Benderitter M. Linard C. (2011). Abdominal gamma-radiation induces an accumulation of function-impaired regulatory T cells in the small intestine. Int. J. Radiat. Oncol. Biol. Phys. 80, 869–876 10.1016/j.ijrobp.2010.12.041 21345609

[B10] Brent G. A. (2010). Environmental exposures and autoimmune thyroid disease. Thyroid 20, 755–761 10.1089/thy.2010.1636 20578899 PMC2935336

[B11] Bruckner L. Gigliotti F. Wright T. Harmsen A. Notter R. H. Chess P. Wang Z. Finkelstein J. (2006). Pneumocystis carinii infection sensitizes lung to radiation-induced injury after syngeneic marrow transplantation: role of CD4+ T cells. Am. J. Physiol. Lung Cell. Mol. Physiol. 290, L1087–L1096 10.1152/ajplung.00441.2005 16399785

[B12] Brush J. Lipnick S. L. Phillips T. Sitko J. McDonald J. T. McBride W. H. (2007). Molecular mechanisms of late normal tissue injury. Semin. Radiat. Oncol. 17, 121–130 10.1016/j.semradonc.2006.11.008 17395042

[B13] Burnette B. C. Liang H. Lee Y. Chlewicki L. Khodarev N. N. Weichselbaum R. R. Fu Y. X. Auh S. L. (2011). The efficacy of radiotherapy relies upon induction of type i interferon-dependent innate and adaptive immunity. Cancer Res. 71, 2488–2496 10.1158/0008-5472.CAN-10-2820 21300764 PMC3070872

[B14] Cao M. Cabrera R. Xu Y. Liu C. Nelson D. (2009). Gamma irradiation alters the phenotype and function of CD4+CD25+ regulatory T cells. Cell Biol. Int. 33, 565–571 10.1016/j.cellbi.2009.02.007 19268553 PMC2683182

[B15] Chen W. Jin W. Hardegen N. Lei K. J. Li L. Marinos N. McGrady G. Wahl S. M. (2003). Conversion of peripheral CD4+CD25- naive T cells to CD4+CD25+ regulatory T cells by TGF-beta induction of transcription factor Foxp3. J. Exp. Med. 198, 1875–1886 10.1084/jem.20030152 14676299 PMC2194145

[B16] Chiang C. S. Liu W. C. Jung S. M. Chen F. H. Wu C. R. McBride W. H. Lee C. C. Hong J. H. (2005). Compartmental responses after thoracic irradiation of mice: strain differences. Int. J. Radiat. Oncol. Biol. Phys. 62, 862–871 10.1016/j.ijrobp.2005.02.037 15936571

[B17] Cronstein B. N. (1994). Adenosine, an endogenous anti-inflammatory agent. J. Appl. Physiol. 76, 5–13 8175547 10.1152/jappl.1994.76.1.5

[B18] Curotto de Lafaille M. A. Lafaille J. J. (2009). Natural and adaptive foxp3+ regulatory T cells: more of the same or a division of labor? Immunity 30, 626–635 10.1016/j.immuni.2009.05.002 19464985

[B19] Curotto de Lafaille M. A. Lino A. C. Kutchukhidze N. Lafaille J. J. (2004). CD25- T cells generate CD25+Foxp3+ regulatory T cells by peripheral expansion. J. Immunol. 173, 7259–7268 15585848 10.4049/jimmunol.173.12.7259

[B20] Curtin J. F. Liu N. Candolfi M. Xiong W. Assi H. Yagiz K. Edwards M. R. Michelsen K. S. Kroeger K. M. Liu C. Muhammad A. K. Clark M. C. Arditi M. Comin-Anduix B. Ribas A. Lowenstein P. R. Castro M. G. (2009). HMGB1 mediates endogenous TLR2 activation and brain tumor regression. PLoS Med. 6:e10 10.1371/journal.pmed.1000010 19143470 PMC2621261

[B21] Eriksen P. L. Kristensen J. E. Knap M. M. (2012). A possible association between coeliac disease and development of radiation pneumonitis. Acta Oncol. [Epub ahead of print]. 10.3109/0284186X.2012.689132 22671574

[B22] Fajardo L. F. (2005). The pathology of ionizing radiation as defined by morphologic patterns. Acta Oncol. 44, 13–22 10.1080/02841860510007440 15848902

[B23] Gallucci S. Lolkema M. Matzinger P. (1999). Natural adjuvants: endogenous activators of dendritic cells. Nat. Med. 5, 1249–1255 10.1038/15200 10545990

[B24] Garnett C. T. Palena C. Chakraborty M. Tsang K. Y. Schlom J. Hodge J. W. (2004). Sublethal irradiation of human tumor cells modulates phenotype resulting in enhanced killing by cytotoxic T lymphocytes. Cancer Res. 64, 7985–7994 10.1158/0008-5472.CAN-04-1525 15520206

[B25] Haribhai D. Williams J. B. Jia S. Nickerson D. Schmitt E. G. Edwards B. Ziegelbauer J. Yassai M. Li S. H. Relland L. M. Wise P. M. Chen A. Zheng Y. Q. Simpson P. M. Gorski J. Salzman N. H. Hessner M. J. Chatila T. A. Williams C. B. (2011). A requisite role for induced regulatory T cells in tolerance based on expanding antigen receptor diversity. Immunity 35, 109–122 10.1016/j.immuni.2011.03.029 21723159 PMC3295638

[B26] Heinzelmann F. Lang P. J. Ottinger H. Faul C. Bethge W. Handgretinger R. Bamberg M. Belka C. (2008). Immunosuppressive total lymphoid irradiation-based reconditioning regimens enable engraftment after graft rejection or graft failure in patients treated with allogeneic hematopoietic stem cell transplantation. Int. J. Radiat. Oncol. Biol. Phys. 70, 523–528 10.1016/j.ijrobp.2007.06.037 17869449

[B27] Hellstrom K. E. Hellstrom I. Kant J. A. Tamerius J. D. (1978). Regression and inhibition of sarcoma growth by interference with a radiosensitive T-cell population. J. Exp. Med. 148, 799–804 308987 10.1084/jem.148.3.799 PMC2184991

[B28] Hofer M. Pospisil M. Znojil V. Vacek A. Weiterova L. Hola J. Vacha J. (2002). Drugs elevating extracellular adenosine promote regeneration of haematopoietic progenitor cells in severely myelosuppressed mice: their comparison and joint effects with the granulocyte colony-stimulating factor. Eur. J. Haematol. 68, 4–11 10.1034/j.1600-0609.2002.00564.x 11995629

[B29] Hong J. H. Jung S. M. Tsao T. C. Wu C. J. Lee C. Y. Chen F. H. Hsu C. H. McBride W. H. Chiang C. S. (2003). Bronchoalveolar lavage and interstitial cells have different roles in radiation-induced lung injury. Int. J. Radiat. Biol. 79, 159–167 10.1080/0955300031000076894 12745880

[B30] Hosek B. Bohacek J. Sikulova J. Pospisil M. Vacek A. (1992). Protection of early cellular damage in 1 Gy-irradiated mice by the elevation of extracellular adenosine. Radiat. Environ. Biophys. 31, 289–297 1438679 10.1007/BF01210209

[B31] Hou B. Xu Z. W. Yang C. W. Gao Y. Zhao S. F. Zhang C. G. (2007). Protective effects of inosine on mice subjected to lethal total-body ionizing irradiation. J. Radiat. Res. (Tokyo) 48, 57–62 17179648 10.1269/jrr.06067

[B32] Huang D. Chen C. Y. Ali Z. Shao L. Shen L. Lockman H. A. Barnewall R. E. Sabourin C. Eestep J. Reichenberg A. Hintz M. Jomaa H. Wang R. Chen Z. W. (2009). Antigen-specific Vgamma2Vdelta2 T effector cells confer homeostatic protection against pneumonic plaque lesions. Proc. Natl. Acad. Sci. U.S.A. 106, 7553–7558 10.1073/pnas.0811250106 19383786 PMC2678605

[B33] Hunig T. (2012). The storm has cleared: lessons from the CD28 superagonist TGN1412 trial. Nat. Rev. Immunol. 12, 317–318 10.1038/nri3192 22487653

[B34] Johnston C. J. Manning C. Hernady E. Reed C. Thurston S. W. Finkelstein J. N. Williams J. P. (2011). Effect of total body irradiation on late lung effects: hidden dangers. Int. J. Radiat. Biol. 87, 902–913 10.3109/09553002.2011.573439 21574903 PMC3296133

[B35] Josefowicz S. Z. Niec R. E. Kim H. Y. Treuting P. Chinen T. Zheng Y. Umetsu D. T. Rudensky A. Y. (2012). Extrathymically generated regulatory T cells control mucosal TH2 inflammation. Nature 482, 395–399 10.1038/nature10772 22318520 PMC3485072

[B36] Kachikwu E. L. Iwamoto K. S. Liao Y. P. Demarco J. J. Agazaryan N. Economou J. S. McBride W. H. Schaue D. (2011). Radiation enhances regulatory T cell representation. Int. J. Radiat. Oncol. Biol. Phys. 81, 1128–1135 10.1016/j.ijrobp.2010.09.034 21093169 PMC3117954

[B37] Kawai T. Akira S. (2011). Toll-like receptors and their crosstalk with other innate receptors in infection and immunity. Immunity 34, 637–650 10.1016/j.immuni.2011.05.006 21616434

[B38] Kusunoki Y. Yamaoka M. Kubo Y. Hayashi T. Kasagi F. Douple E. B. Nakachi K. (2010). T-cell immunosenescence and inflammatory response in atomic bomb survivors. Radiat. Res. 174, 870–876 10.1667/RR1847.1 21128811

[B39] Laetsch B. Hofer T. Lombriser N. Lautenschlager S. (2011). Irradiation-induced morphea: x-rays as triggers of autoimmunity. Dermatology 223, 9–12 10.1159/000330324 21865672

[B40] Linard C. Billiard F. Benderitter M. (2012). Intestinal irradiation and fibrosis in a Th1-deficient environment. Int. J. Radiat. Oncol. Biol. Phys. 84, 266–273 10.1016/j.ijrobp.2011.11.027 22336200

[B41] Loor F. Jachez B. Montecino-Rodriguez E. Klein A. S. Kuntz L. Pflumio F. Fonteneau P. Illinger D. (1988). Radiation therapy of spontaneous autoimmunity: a review of mouse models. Int. J. Radiat. Biol. Relat. Stud. Phys. Chem. Med. 53, 119–136 2892809 10.1080/09553008814550481

[B42] Lorimore S. A. Coates P. J. Scobie G. E. Milne G. Wright E. G. (2001). Inflammatory-type responses after exposure to ionizing radiation *in vivo*: a mechanism for radiation-induced bystander effects? Oncogene 20, 7085–7095 10.1038/sj.onc.1204903 11704832

[B43] Lotze M. T. Zeh H. J. Rubartelli A. Sparvero L. J. Amoscato A. A. Washburn N. R. Devera M. E. Liang X. Tor M. Billiar T. (2007). The grateful dead: damage-associated molecular pattern molecules and reduction/oxidation regulate immunity. Immunol. Rev. 220, 60–81 10.1111/j.1600-065X.2007.00579.x 17979840

[B44] Lugade A. A. Moran J. P. Gerber S. A. Rose R. C. Frelinger J. G. Lord E. M. (2005). Local radiation therapy of B16 melanoma tumors increases the generation of tumor antigen-specific effector cells that traffic to the tumor. J. Immunol. 174, 7516–7523 15944250 10.4049/jimmunol.174.12.7516

[B45] Lugade A. A. Sorensen E. W. Gerber S. A. Moran J. P. Frelinger J. G. Lord E. M. (2008). Radiation-induced IFN-gamma production within the tumor microenvironment influences antitumor immunity. J. Immunol. 180, 3132–3139 18292536 10.4049/jimmunol.180.5.3132

[B46] MacLennan I. C. Kay H. E. (1978). Analysis of treatment in childhood leukemia. IV. The critical association between dose fractionation and immunosuppression induced by cranial irradiation. Cancer 41, 108–111 272224 10.1002/1097-0142(197801)41:1<108::aid-cncr2820410116>3.0.co;2-z

[B47] Mandapathil M. Szczepanski M. J. Szajnik M. Ren J. Lenzner D. E. Jackson E. K. Gorelik E. Lang S. Johnson J. T. Whiteside T. L. (2009). Increased ectonucleotidase expression and activity in regulatory T cells of patients with head and neck cancer. Clin. Cancer Res. 15, 6348–6357 10.1158/1078-0432.CCR-09-1143 19825957 PMC2763335

[B48] Martin M. Lefaix J. Delanian S. (2000). TGF-beta1 and radiation fibrosis: a master switch and a specific therapeutic target? Int. J. Radiat. Oncol. Biol. Phys. 47, 277–290 10802350 10.1016/s0360-3016(00)00435-1

[B49] Matsumura S. Wang B. Kawashima N. Braunstein S. Badura M. Cameron T. O. Babb J. S. Schneider R. J. Formenti S. C. Dustin M. L. Demaria S. (2008). Radiation-induced CXCL16 release by breast cancer cells attracts effector T cells. J. Immunol. 181, 3099–3107 18713980 10.4049/jimmunol.181.5.3099 PMC2587101

[B50] Matzinger P. (2002). The danger model: a renewed sense of self. Science 296, 301–305 10.2353/ajpath.2008.070563 11951032

[B51] Matzinger P. Kamala T. (2011). Tissue-based class control: the other side of tolerance. Nat. Rev. Immunol. 11, 221–230 10.1038/nri2940 21350581

[B52] McBride W. H. Chiang C. S. Olson J. L. Wang C. C. Hong J. H. Pajonk F. Dougherty G. J. Iwamoto K. S. Pervan M. Liao Y. P. (2004). A sense of danger from radiation. Radiat. Res. 162, 1–19 15222781 10.1667/rr3196

[B53] McBride W. H. Vegesna V. (1997). Role of the thymus in radiation-induced lung damage after bone marrow transplantation. Radiat. Res. 147, 501–505 9092932

[B54] McBride W. H. Vegesna V. (2000). The role of T-cells in radiation pneumonitis after bone marrow transplantation. Int. J. Radiat. Biol. 76, 517–521 10815632 10.1080/095530000138529

[B55] Miller Y. I. Choi S. H. Wiesner P. Fang L. Harkewicz R. Hartvigsen K. Boullier A. Gonen A. Diehl C. J. Que X. Montano E. Shaw P. X. Tsimikas S. Binder C. J. Witztum J. L. (2011). Oxidation-specific epitopes are danger-associated molecular patterns recognized by pattern recognition receptors of innate immunity. Circ. Res. 108, 235–248 10.1161/CIRCRESAHA.110.223875 21252151 PMC3075542

[B56] Moravan M. J. Olschowka J. A. Williams J. P. O'Banion M. K. (2011). Cranial irradiation leads to acute and persistent neuroinflammation with delayed increases in T-cell infiltration and CD11c expression in C57BL/6 mouse brain. Radiat. Res. 176, 459–473 21787181 10.1667/rr2587.1 PMC3191189

[B57] Morel A. Fernandez N. de La Coste A. Haddada H. Viguier M. Polla B. S. Antoine B. Kahn A. (1998). Gamma-ray irradiation induces B7.1 costimulatory molecule neoexpression in various murine tumor cells. Cancer Immunol. Immunother. 46, 277–282 9690456 10.1007/s002620050488 PMC11037350

[B58] Mosley R. L. Klein J. R. (1992). Repopulation kinetics of intestinal intraepithelial lymphocytes in murine bone marrow radiation chimeras. Transplantation 53, 868–874 1533071 10.1097/00007890-199204000-00030

[B59] Nador R. G. Hongo D. Baker J. Yao Z. Strober S. (2010). The changed balance of regulatory and naive T cells promotes tolerance after TLI and anti-T-cell antibody conditioning. Am. J. Transplant. 10, 262–272 10.1111/j.1600-6143.2009.02942.x 20041865 PMC2886014

[B60] Nagayama Y. Ichikawa T. Saitoh O. Abiru N. (2009). Induction of late-onset spontaneous autoimmune thyroiditis by a single low-dose irradiation in thyroiditis-prone non-obese diabetic-H2h4 mice. J. Radiat. Res. 50, 573–577 19755803 10.1269/jrr.09067

[B61] Nakatsukasa H. Tsukimoto M. Tokunaga A. Kojima S. (2010). Repeated gamma irradiation attenuates collagen-induced arthritis via up-regulation of regulatory T cells but not by damaging lymphocytes directly. Radiat. Res. 174, 313–324 10.1667/RR2121.1 20726718

[B62] Nakayama Y. Makino S. Fukuda Y. Min K. Y. Shimizu A. Ohsawa N. (1996). Activation of lavage lymphocytes in lung injuries caused by radiotherapy for lung cancer. Int. J. Radiat. Oncol. Biol. Phys. 34, 459–467 8567349 10.1016/0360-3016(95)02101-9

[B63] North R. J. (1986). Radiation-induced, immunologically mediated regression of an established tumor as an example of successful therapeutic immunomanipulation. Preferential elimination of suppressor T cells allows sustained production of effector T cells. J. Exp. Med. 164, 1652–1666 2945892 10.1084/jem.164.5.1652 PMC2188446

[B64] Pan F. Barbi J. Pardoll D. M. (2012). Hypoxia-inducible factor 1: a link between metabolism and T cell differentiation and a potential therapeutic target. Oncoimmunology 1, 510–515 22754770 10.4161/onci.19457 PMC3382896

[B65] Pelus L. M. Fukuda S. (2008). Chemokine-mobilized adult stem cells; defining a better hematopoietic graft. Leukemia 22, 466–473 10.1038/sj.leu.2405021 17972941 PMC2814589

[B66] Peterson R. A. (2012). Regulatory T-cells: diverse phenotypes integral to immune homeostasis and suppression. Toxicol. Pathol. 40, 186–204 10.1177/0192623311430693 22222887

[B67] Pospisil M. Hofer M. Netikova J. Pipalova I. Vacek A. Bartonickova A. Volenec K. (1993). Elevation of extracellular adenosine induces radioprotective effects in mice. Radiat. Res. 134, 323–330 8316625

[B68] Pospisil M. Hofer M. Znojil V. Netikova J. Vacha J. Hola J. Vacek A. (1998). Granulocyte colony-stimulating factor and drugs elevating extracellular adenosine synergize to enhance haematopoietic reconstitution in irradiated mice. Eur. J. Haematol. 60, 172–180 9548416 10.1111/j.1600-0609.1998.tb01019.x

[B69] Qu Y. Jin S. Zhang A. Zhang B. Shi X. Wang J. Zhao Y. (2010). Gamma-ray resistance of regulatory CD4+CD25+Foxp3+ T cells in mice. Radiat. Res. 173, 148–157 10.1667/RR0978.1 20095846

[B70] Radhakrishnan S. Cabrera R. Schenk E. L. Nava-Parada P. Bell M. P. Van Keulen V. P. Marler R. J. Felts S. J. Pease L. R. (2008). Reprogrammed FoxP3+ T regulatory cells become IL-17+ antigen-specific autoimmune effectors *in vitro* and *in vivo*. J. Immunol. 181, 3137–3147 18713984 10.4049/jimmunol.181.5.3137 PMC2582200

[B71] Reits E. A. Hodge J. W. Herberts C. A. Groothuis T. A. Chakraborty M. Wansley E. K. Camphausen K. Luiten R. M. de Ru A. H. Neijssen J. Griekspoor A. Mesman E. Verreck F. A. Spits H. Schlom J. van Veelen P. Neefjes J. J. (2006). Radiation modulates the peptide repertoire, enhances MHC class I expression, and induces successful antitumor immunotherapy. J. Exp. Med. 203, 1259–1271 10.1084/jem.20052494 16636135 PMC3212727

[B72] Rosenblum M. D. Gratz I. K. Paw J. S. Lee K. Marshak-Rothstein A. Abbas A. K. (2011). Response to self antigen imprints regulatory memory in tissues. Nature 480, 538–542 10.1038/nature10664 22121024 PMC3263357

[B73] Sakaguchi N. Miyai K. Sakaguchi S. (1994). Ionizing radiation and autoimmunity. Induction of autoimmune disease in mice by high dose fractionated total lymphoid irradiation and its prevention by inoculating normal T cells. J. Immunol. 152, 2586–2595 8133066

[B74] Sakaguchi S. (2004). Naturally arising CD4+ regulatory t cells for immunologic self-tolerance and negative control of immune responses. Annu. Rev. Immunol. 22, 531–562 10.1146/annurev.immunol.21.120601.141122 15032588

[B75] Sakaguchi S. (2005). Naturally arising Foxp3-expressing CD25+CD4+ regulatory T cells in immunological tolerance to self and non-self. Nat. Immunol. 6, 345–352 10.1038/ni1178 15785760

[B76] Santin A. D. Hiserodt J. C. Fruehauf J. DiSaia P. J. Pecorelli S. Granger G. A. (1996). Effects of irradiation on the expression of surface antigens in human ovarian cancer. Gynecol. Oncol. 60, 468–474 10.1006/gyno.1996.0075 8774659

[B77] Sato Y. Goto Y. Narita N. Hoon D. S. (2009). Cancer cells expressing toll-like receptors and the tumor microenvironment. Cancer Microenviron. 2(Suppl. 1), 205–214 10.1007/s12307-009-0022-y 19685283 PMC2756339

[B79] Schaue D. Kachikwu E. L. McBride W. H. (2012). Cytokines in radiobiological responses: a review. Radiat. Res. (in press). 10.1667/RR3031.1 PMC3723384 23106210

[B78] Schaue D. McBride W. H. (2010). Links between innate immunity and normal tissue radiobiology. Radiat. Res. 173, 406–417 10.1667/RR1931.1 20334512 PMC2865470

[B80] Seo A. Ishikawa F. Nakano H. Nakazaki H. Kobayashi K. Kakiuchi T. (1999). Enhancement of B7-1 (CD80) expression on B-lymphoma cells by irradiation. Immunology 96, 642–648 10.1046/j.1365-2567.1999.00720.x 10233753 PMC2326785

[B81] Shankar G. Scott Bryson J. Darrell Jennings C. Kaplan A. M. Cohen D. A. (1999). Idiopathic pneumonia syndrome after allogeneic bone marrow transplantation in mice. Role of pretransplant radiation conditioning. Am. J. Respir. Cell Mol. Biol. 20, 1116–1124 10340930 10.1165/ajrcmb.20.6.3455

[B82] Shi J. Ge M. Lu S. Li X. Shao Y. Huang J. Huang Z. Zhang J. Nie N. Zheng Y. (2012). Intrinsic impairment of CD4+CD25+ regulatory T cells in acquired aplastic anemia. Blood 120, 1624–1632 10.1182/blood-2011-11-390708 22797698

[B83] Shi Y. Evans J. E. Rock K. L. (2003). Molecular identification of a danger signal that alerts the immune system to dying cells. Nature 425, 516–521 10.1038/nature01991 14520412

[B84] Steinman R. M. Hawiger D. Nussenzweig M. C. (2003). Tolerogenic dendritic cells. Annu. Rev. Immunol. 21, 685–711 10.1146/annurev.immunol.21.120601.141040 12615891

[B85] Takahashi H. Kanno T. Nakayamada S. Hirahara K. Sciume G. Muljo S. A. Kuchen S. Casellas R. Wei L. Kanno Y. O'Shea J. J. (2012). TGF-beta and retinoic acid induce the microRNA miR-10a, which targets Bcl-6 and constrains the plasticity of helper T cells. Nat. Immunol. 13, 587–595 10.1038/ni.2286 22544395 PMC3499969

[B86] Teymoortash A. Simolka N. Schrader C. Tiemann M. Werner J. A. (2005). Lymphocyte subsets in irradiation-induced sialadenitis of the submandibular gland. Histopathology 47, 493–500 10.1111/j.1365-2559.2005.02256.x 16241997

[B87] Tilkin A. F. Schaaf-Lafontaine N. Van Acker A. Boccadoro M. Urbain J. (1981). Reduced tumor growth after low-dose irradiation or immunization against blastic suppressor T cells. Proc. Natl. Acad. Sci. U.S.A. 78, 1809–1812 6453350 10.1073/pnas.78.3.1809 PMC319224

[B88] Toma C. L. Serbescu A. Alexe M. Cervis L. Ionita D. Bogdan M. A. (2010). The bronchoalveolar lavage pattern in radiation pneumonitis secondary to radiotherapy for breast cancer. Maedica (Buchar) 5, 250–257 21977166 PMC3152839

[B89] Tsakiri N. Papadopoulos D. Denis M. C. Mitsikostas D. D. Kollias G. (2012). TNFR2 on non-haematopoietic cells is required for Foxp3+ Treg-cell function and disease suppression in EAE. Eur. J. Immunol. 42, 403–412 10.1002/eji.201141659 22105853

[B90] Tsukimoto M. Nakatsukasa H. Sugawara K. Yamashita K. Kojima S. (2008). Repeated 0.5-Gy gamma irradiation attenuates experimental autoimmune encephalomyelitis with up-regu-lation of regulatory T cells and suppression of IL17 production. Radiat. Res. 170, 429–436 19024649 10.1667/rr1352.1

[B91] Tyurina Y. Y. Tyurin V. A. Kapralova V. I. Wasserloos K. Mosher M. Epperly M. W. Greenberger J. S. Pitt B. R. Kagan V. E. (2011). Oxidative lipidomics of gamma-radiation-induced lung injury: mass spectrometric characterization of cardiolipin and phosphatidylserine peroxidation. Radiat. Res. 175, 610–621 10.1667/RR2297.1 21338246 PMC3321728

[B92] Urbieta M. Barao I. Jones M. Jurecic R. Panoskaltsis-Mortari A. Blazar B. R. Murphy W. J. Levy R. B. (2010). Hematopoietic progenitor cell regulation by CD4+CD25+ T cells. Blood 115, 4934–4943 10.1182/blood-2009-04-218826 20200356 PMC2890189

[B93] van Wijk F. Roord S. T. Vastert B. de Kleer I. Wulffraat N. Prakken B. J. (2008). Regulatory T cells in autologous stem cell transplantation for autoimmune disease. Autoimmunity 41, 585–591 10.1080/08916930802200182 18958758

[B94] Waer M. Ang K. K. Van der Schueren E. Vandeputte M. (1984). Influence of radiation field and fractionation schedule of total lymphoid irradiation (TLI) on the induction of suppressor cells and stable chimerism after bone marrow transplantation in mice. J. Immunol. 132, 985–990 6228603

[B95] Walker L. S. (2004). CD4+ CD25+ Treg: divide and rule? Immunology 111, 129–137 10.1111/j.0019-2805.2003.01788.x 15027897 PMC1782405

[B96] Weigensberg M. Morecki S. Weiss L. Fuks Z. Slavin S. (1984). Suppression of cell-mediated immune responses after total lymphoid irradiation (TLI). I. Characterization of suppressor cells of the mixed lymphocyte reaction. J. Immunol. 132, 971–978 6140288

[B97] Weng L. Williams R. O. Vieira P. L. Screaton G. Feldmann M. Dazzi F. (2010). The therapeutic activity of low-dose irradiation on experimental arthritis depends on the induction of endogenous regulatory T cell activity. Ann. Rheum. Dis. 69, 1519–1526 10.1136/ard.2009.121111 20498214

[B98] Wilson J. M. Kurtz C. C. Black S. G. Ross W. G. Alam M. S. Linden J. Ernst P. B. (2011). The A2B adenosine receptor promotes Th17 differentiation via stimulation of dendritic cell IL-6. J. Immunol. 186, 6746–6752 10.4049/jimmunol.1100117 21593380 PMC3365485

[B99] Yan R. Peters L. J. Travis E. L. (1991). Cyclophosphamide 24 hours before or after total body irradiation: effects on lung and bone marrow. Radiother. Oncol. 21, 149–156 10.1016/0167-8140(91)90031-B 1924849

[B100] Yao Z. Jones J. Kohrt H. Strober S. (2011). Selective resistance of CD44hi T cells to p53-dependent cell death results in persistence of immunologic memory after total body irradiation. J. Immunol. 187, 4100–4108 10.4049/jimmunol.1101141 21930972 PMC3186844

[B101] Yoshii Y. (2008). Pathological review of late cerebral radionecrosis. Brain Tumor Pathol. 25, 51–58 10.1007/s10014-008-0233-9 18987829

[B102] Zarek P. E. Huang C. T. Lutz E. R. Kowalski J. Horton M. R. Linden J. Drake C. G. Powell J. D. (2008). A2A receptor signaling promotes peripheral tolerance by inducing T-cell anergy and the generation of adaptive regulatory T cells. Blood 111, 251–259 10.1182/blood-2007-03-081646 17909080 PMC2200810

